# Transactional Linkages Between Parenting Behaviors and Child Executive Functions and Self-Regulation from Early Childhood to Adolescence

**DOI:** 10.17505/jpor.2024.26261

**Published:** 2024-05-23

**Authors:** Atika Khurana, Heather Leonard, Laura Michaelson, Derek Kosty

**Affiliations:** 1 College of Education, University of Oregon, USA; 2 American Institutes for Research, USA; 3 Prevention Science Institute, University of Oregon, USA

**Keywords:** parenting, executive function, working memory, inhibitory control, self-regulation, early childhood, adolescence

## Abstract

Research on the development of executive functions (EFs) and self-regulation (SR) has focused heavily on the early childhood years, when these abilities first emerge. Less is known in comparison about how these abilities develop through adolescence, and how contextual factors, such as parenting, influence their development in later years. Using longitudinal data from the Study of Early Child Care and Youth Development (SECCYD), we used random intercept cross-lagged panel modeling (RI-CLPM) to examine the bidirectional linkages between three parenting behaviors (i.e., autonomy support, supportive presence, hostility), child EFs (i.e., working memory, inhibitory control) and child SR outcomes, from early childhood to adolescence. Parenting in early childhood was significantly associated with change in child EFs from early to middle childhood, but not from middle childhood to adolescence. Specifically, greater autonomy support in early childhood was associated with stronger child working memory and inhibitory control in middle childhood; greater supportive presence in early childhood was associated with stronger child working memory in middle childhood; and higher rates of hostility in early childhood were associated with weaker child inhibitory skills in middle childhood. Reciprocal effects of child EF and SR on parenting were also observed. Specifically, stronger child inhibitory control in early childhood was associated with less hostility in middle childhood, and stronger child self-regulation in middle childhood was associated with greater supportive presence in adolescence. Accounting for lagged and stability effects, there was significant residual covariance between parenting behaviors and child SR in adolescence, suggesting that parenting continues to be associated with the development of SR skills through adolescence. Understanding reciprocal linkages between parenting and child EF/SR through adolescence is critical in developing targeted parenting interventions beyond early childhood to improve children’s outcomes.

## Introduction

Self-regulation (SR) – a multi-dimensional construct that encompasses voluntary regulation of one’s thoughts, emotions, and behaviors in the service of a goal (McClelland et al., 2010) – is a well-established predictor of long-term health and well-being outcomes (Moffitt et al., 2011). The ability to self-regulate relies on executive functions (EFs), which are higher-order cognitive functions that enable the top-down regulation of behavioral urges, thoughts, and emotions in a flexible, goal-directed manner. Within a broader, unified framework (Blair & Ku, 2022), SR is conceptualized to include *all* the systems and processes by which an individual guides their behavior towards desired end states (Gillebaart, 2018). In this study, we focus on one particular aspect of SR, sometimes referred to as “self-control”, by which EFs enable the top-down control of pre-potent responses (Cole et al., 2019). There are three core EFs: *working memory* (i.e., the ability to hold information in mind and operate on it simultaneously), *inhibitory control* (i.e., the ability to inhibit automatic reactions and manage distractions), and *cognitive flexibility* (i.e., the ability to focus and shift attentional focus; Miyake et al., 2000). Both the capacity to self-regulate and the underlying EFs undergo rapid development during the early childhood years (Diamond, 2013), with continued growth and plasticity through adolescence and early adulthood (Geidd et al., 1999; Luna et al., 2015).

The development of SR is a dynamic process and happens within relational contexts. In younger years as caregivers respond sensitively to the child’s needs, children develop attention control and emotional and behavioral regulation skills, which set the stage for the development of EFs. Over time, and through continued maturation of EFs, children are able to regulate their own emotions and behaviors without external support and can do so consistently across different contexts (Blair & Ku, 2022; Cole et al., 2019). Given their salience as transdiagnostic predictors of long-term health and wellbeing outcomes, as well as their relative malleability to environmental inputs (Diamond & Lee, 2011), EFs and SR have received considerable research attention (Robson et al., 2020) and have been the target of intervention efforts (Bradshaw et al., 2012).

Most prior work examining the development of EFs and SR has focused on early childhood years (birth – 6 years) when these abilities first emerge and undergo rapid development (Rosanbalm & Murray, 2017). We know much less in comparison about the development of these skills during later years, and further, how contextual factors such as parenting influence the development of these skills through later childhood and adolescence. Adolescence is a unique window of neuroplasticity when EF and SR capacities are refined and shaped by interactions in social contexts (Murty et al., 2016). Understanding parental influences on the development of these abilities through adolescence is critical in developing targeted parenting interventions beyond early childhood to improve children’s EFs and SR (Rosanbalm & Murray, 2017). The present study examined the impact of parenting behaviors on the development of child EFs and SR, from early childhood through adolescence, using a developmental-transactional framework that accounts for the dynamic, bidirectional nature of these effects (Belsky et al., 2007, Wang et al., 2013). Although a few studies have documented parenting effects on SR in adolescence (Fosco et al., 2013, King et al., 2013, Li et al., 2019), no study to date has examined transactional linkages between parenting behaviors and child EFs and SR systematically from early childhood through adolescence.

Further, most prior work examining parenting effects on child EFs and SR has focused on global parenting constructs (e.g., positive parenting; Rochette & Bernier, 2014) rather than specific parenting behaviors, even though there is evidence that specific parenting behaviors (e.g., autonomy support) can have stronger effects on child SR than other parenting behaviors (e.g., maternal warmth; Bindman et al., 2015). There also has been limited examination of the relative magnitude and timing of these effects. For instance, some parenting behaviors (e.g., autonomy support) can have stronger effects on child EFs as compared to child SR (Hughes & Devine, 2019), especially during younger years (Belsky et al., 2007). This is consistent with other studies documenting stronger parenting effects on child EFs as compared to child SR, particularly during early childhood years (Valcan et al., 2018). Thus, besides the relative magnitude of specific parenting effects across EFs and SR, the timing of these effects can also vary.

Children are also active agents in their social interactions, and thus can impact the parenting they receive. Child effects on parenting behaviors tend to be more pronounced in later years (Wang et al., 2013) and have more consistently been observed in the case of child temperament (Kiff et al., 2011) and behavioral self-regulation (e.g., externalizing behaviors; Wang et al., 2013) influencing parenting. The reciprocal effects of child EFs on parenting behaviors are less well documented and have mostly been reported during preschool/ early childhood years (Belsky et al., 2007; Merz et al., 2017). To our knowledge, no study to date has examined bidirectional effects between specific parenting behaviors and child EFs and SR in a longitudinal sample from early childhood to adolescence, though some studies have examined the reciprocal effects of parenting behaviors on a related dimension of effortful control (e.g., Tiberio et al., 2016).

### Parenting Effects on Child Executive Functions and Self-regulation

For this study, we focused on three specific parenting behaviors: autonomy support, supportive presence, and hostility, given prior evidence of their associations with child EFs and SR (e.g., Bernier et al., 2010; Bindman et al., 2015; Kok et al., 2022; Lucassen et al., 2015; Wang et al., 2013). In the following section, we review studies that have found associations between these specific parenting behaviors and child EFs and SR. Because we tested our research questions using data from the Early Child Care and Youth Development (SECCYD) study, previous findings using the same dataset are noted as such. Although all the SECCYD-based studies reviewed below utilized the observational coding of motherchild interactions, they focused on a global construct of maternal sensitivity (which included dimensions of autonomy support, supportive presence, and inverse of hostility) in relation to child outcomes (Wang et al., 2013). The construct of maternal sensitivity was significantly associated with both child EFs (Belsky et al., 2007; Conway, 2020) and SR (Conway, 2020). Studies with other/non-SECCYD samples have similarly found significant associations between global parenting constructs and child EFs/SR outcomes (e.g., Sulik et al., 2015). However, given the importance of understanding the effects of specific parenting behaviors in developing targeted interventions, we take a more fine-grained approach to examining the effects of the three parenting behaviors commonly included in the maternal sensitivity construct (i.e., autonomy support, supportive presence, and hostility). We also tested the effects of these parenting behaviors on child EFs and SR outcomes separately. Even though EFs and SR measures tend to be correlated, they are assessing different constructs. EFs represent internal cognitive processes that enable SR, which, in turn, is a broader ability to regulate thoughts, emotions, and behaviors in a goal-directed manner (McClelland et al., 2010). Prior evidence regarding the associations between the three parenting behaviors and child EFs/SR is reviewed next.

*Autonomy support* reflects parent’s respect and support of the child’s individuality, allowing the child to actively participate in problem-solving and completing tasks without intrusiveness, thus helping them achieve a sense of accomplishment and develop self-efficacy (Bernier et al., 2010). In a study using the SECCYD data, high levels of maternal autonomy support assessed during the first three years of child’s life predicted stronger EFs at 54 months, which in turn was predictive of greater academic achievement in elementary (Grades 1, 3, and 5) and high school (child age 15) years (Bindman et al., 2015). The effect of maternal autonomy support on child EFs remained significant even when controlling for other parenting dimensions such as maternal warmth and cognitive stimulation.

In another sample of 78 mother-child dyads (82% Caucasian) recruited from a large Canadian metropolitan area, maternal autonomy support at child age 15 months, and the average maternal autonomy support at 15 months and 3 years was positively associated with child EF at age 3 (MatteGagne et al., 2015). Using the same sample, Bernier and colleagues (2010) compared the effects of maternal sensitivity, mind-mindedness, and autonomy support at child age 15 months on child EFs at 18 months and 26 months and found that only maternal autonomy support had significant associations with child EFs accounting for control variables of maternal education and child general cognitive ability.

*Supportive presence*, which reflects parents’ positive regard for their child and encouragement of their efforts, has also been linked to child EFs and SR (Bradley & Corwyn, 2008; Landry & Smith, 2010). Supportive parenting facilitates the development of children’s regulatory ability, as the external support and regulation provided by the caregiver gradually becomes internalized in a safe and positive relational context (Fay-Stammbach et al., 2014). When parents are positively supportive of their child’s needs, the child develops trust, security, and predictability, which facilitates behavioral and emotional regulation. Supportive parental responses can help children not get over-aroused in distressing situations and learn to better regulate their emotions and behaviors through modeling and secure attachment relationships (Spinrad et al., 2007). In a longitudinal study of boys from low SES backgrounds, maternal positive support assessed in infancy was associated with children’s attentional focus and flexibility at age 3.5 years and better self-control at school entry (Gilliom et al., 2002). Positive effects of maternal supportive presence on child SR has also been observed in adolescent samples (Moilanen & Rambo-Hernandez, 2017).

*Hostility*, which reflects parental rejection and blaming the child for mistakes, has been negatively linked to child’s EF and SR development. Hostility is commonly viewed as the opposite of parental warmth and is found to undermine the development of child SR (Fay-Stammbach et al., 2014). Specifically, hostile forms of parenting model dysregulated behaviors for the child and make it difficult for the child to engage their EFs in the context of heightened negative affect, thus compromising their ability to develop self-regulatory skills (Moilanen et al., 2018). Hostile parenting can also interfere with the development of a secure attachment with the parent/caregiver. Consequently, the child is less likely to internalize and comply with parental behavioral expectations. Additionally, hostile parenting can operate as risk factor for coercive parent-child interaction patterns where child difficulties with SR and parents’ harsh and controlling responses mutually reinforce each other leading to further exacerbation of child behavioral problems (Scaramella & Leve, 2004). The negative effects of hostile parenting on child EFs have been documented in samples as young as 3 years of age (Blair et al., 2011), with several studies documenting longitudinal associations between hostile parenting and EF deficits in early childhood (e.g., Cuevas et al., 2014; Lam et al., 2018; Zhang & Li, 2022) and middle childhood years (Halse et al., 2019).

### Timing of Effects

The effects of different parenting behaviors on child EFs and SR are found to vary across the developmental periods (Bradley et al., 2017). For instance, supportive presence and (lack of) hostility tend to matter more during the younger years when children are establishing attachments (Belsky et al., 2007; Wang et al., 2013), but autonomy support can be relevant even during adolescence as children develop greater independence from parents. In a study using SECCYD data, the effect of a global parenting composite of maternal sensitivity on child EFs was significant at 18 months but diminished by 26 months, whereas the effects of specific dimensions like autonomy support remained significant across the 26 months follow-up (Bernier et al., 2010). Other studies using the SECCYD dataset found similar tapering of parenting effects over time, but there is individual variability across different parenting behaviors (Belsky et al., 2007). As such, it is important to examine effects of specific parenting behaviors over time to test if the effects wane across childhood. This information would also help in providing targeting interventions at developmental time points when they are more likely to be effective.

### Child Evocative Effects

Appropriately testing developmental timing of parenting effects also requires attention to the directionality of effects and understanding to what extent child behavior influences parenting behaviors. Child effects on parenting behaviors have been studied more extensively in younger children than in adolescents (Belsky et al., 2007) even though past work suggests that such evocative effects are likely to become stronger as children age (Wang et al., 2013). For instance, a SECCYD study found that the association between maternal sensitivity and child externalizing behaviors from ages 4 to 11 years was driven by child effects on maternal behaviors and not vice versa (Wang et al., 2013). In our models, we tested evocative effects of child EFs and SR on parenting behaviors from early childhood to adolescence.

### Mechanisms of Effects

Even though EFs and SR are interrelated, they represent distinct constructs with specific developmental time frames and unique associations with environmental inputs. For instance, studies have found that child EFs develop earlier in development than child SR, and are more sensitive to environmental impacts during younger years than in later years (Bernier et al., 2010; Valcan et al., 2018). In comparison, environmental (including intervention) effects on child SR have been documented even in adolescent years (Fosco et al., 2013; King et al., 2013). Further, since EFs are thought to underlie self-regulated behavior, it can be expected that some of the effect of parenting behaviors on child SR might be mediated by child EFs, and possibly emerge in later years. Prior work has found more consistent and stronger effects of early parenting on child EFs than child SR (e.g., Belsky et al., 2007). This may be because EFs are thought to underlie SR (McClelland et al., 2010), and thus may mediate the effects of parenting on child SR. We tested this putative mechanism of influence by including indirect effect pathways through child EFs in our models.

Although not specific to SR, there is evidence of how parenting effects on long-term academic outcomes and behavioral dysregulation (e.g., externalizing behaviors) are mediated by child EFs. For instance, using the SECCYD data, Belsky and associates found that the effect of maternal sensitivity on child externalizing behaviors was mediated by child attentional control (Belsky et al., 2007). Bindman and colleagues similarly reported that the effect of autonomysupportive parenting during the first 3 years of life on a child’s academic achievement in elementary and middle school was partially mediated by child EFs (Bindman et al., 2015). There is also evidence that parenting behaviors affect adolescent SR development through their effects on attention regulation and EF development during younger years (Berthelsen et al., 2017). Using a different longitudinal sample, Eisenberg and colleagues found that the effect of parental warmth and positive affect (assessed at age 9) on adolescent externalizing behaviors (assessed six years later) was mediated by the effects of these parenting behaviors on child effortful control (Eisenberg et al., 2005). These findings suggest that the effects of parenting behaviors on child SR may be mediated by child EFs.

Further, the timing and mechanisms of these indirect effects can vary. For instance, early deficits in child EFs can worsen if the parenting context is not supportive, resulting in downstream effects on the ability to self-regulate (Gueron-Sela et al., 2018). It is also possible that early weakness in child EFs elicits negative parenting, which further exacerbates EF and SR deficits. Negative parenting behaviors (e.g., hostility) can also make it difficult for the child to engage EFs in the context of heightened negative affect (Hughes & Ensor, 2006), thus compromising the ability to develop self-regulatory skills. In contrast, when parents are sensitive to their child’s needs, the child develops trust, security, and predictability, which facilitates attentional regulation (Brody & Ge, 2001). As such, it is important to test these indirect effects over time using transactional models.

### Isolating Adolescence-Specific Associations between Parenting and Child Outcomes

Parenting behaviors have been linked to child EF and SR development in early childhood years, but parenting effects on these outcomes are not well examined in adolescent years. Adolescence, in particular mid-adolescence, is a critical developmental period marked by a surge in sensation seeking (Khurana et al., 2018) and risk-taking tendencies as well as a peak in parent-adolescent conflicts (Collins & Steinberg, 2007). Social interactions (such as parenting effects) can have a significant effect on developing EFs and SR abilities during adolescence. Adolescents who have stronger EFs and SR are less likely to engage in impulsive forms of risk taking (Khurana et al., 2015, 2017) and have better long-term health and well-being outcomes (Michaelson & Munakata, 2020; Moffitt et al., 2011). Testing parenting effects from early childhood through adolescence can reveal critical information about how individual differences in adolescent EFs and SR are shaped by parenting behaviors – concurrently (i.e., parenting during adolescence), as early enduring effects (i.e., early parenting effects on child EFs and SR that remain stable over time), lagged effects (i.e., parenting effects on child EFs and SR from one time point to the next), and late emerging effects (early parenting effects on child EFs and SR that manifest in adolescence). This comprehensive understanding of parenting effects on child EFs and SR across development will help with designing targeted parentingbased interventions that can be delivered at optimal developmental stages to have maximum impact.

### Present Study

We used longitudinal data from the SECCYD study to examine population-level transactional linkages between three specific maternal parenting behaviors (i.e., autonomy support, supportive presence, hostility) and two child EFs (i.e., working memory, inhibitory control) and child SR from early childhood-to- middle childhood-to- adolescence. The first research objective examined the unique effects of three specific parenting behaviors on child EFs and SR across development as lagged effects, early enduring effects, and late emerging effects. We hypothesized that the effects of autonomy support on child EFs and SR would be stronger than the effects of supportive presence and hostility (Bernier et al., 2010; Bindman et al., 2015). In terms of timing of effects, autonomy support was expected to have similar magnitude of effects from early childhood to adolescence, whereas the effects of supportive presence and hostility were expected to decline with age as children become better at regulating their emotions and behaviors (Bernier et al., 2010).

The second research objective assessed reciprocal effects of child EFs and SR on parenting behaviors across development, and the developmental timing of these effects. We expected child EFs and SR to have reciprocal effects on parenting behaviors (Wesarg-Menzel et al., 2023), such that stronger EFs and SR would elicit more positive parenting behaviors and vice versa, with the effects being more pronounced during middle childhood and adolescence than early childhood (Wang et al., 2013).

The third research objective explored the mechanisms of these effects, in particular if the effect of parenting on child SR was mediated by child EFs. We hypothesized that parenting behaviors will have an indirect effect on child SR, mediated by child EFs.

The fourth and final research objective tested the presence of residual covariances between parenting behaviors and child EFs and SR during adolescence. We expected to find significant residual covariances between parenting behaviors and child EFs and SR in adolescence, accounting for lagged effects, early enduring effects, and late emerging effects. Our study hypotheses were pre-registered in 2021 (https://osf.io/86ntb).

A significant contribution of this study is that it examines the transactional linkages between parenting behaviors and child EFs and SR across the entire developmental time frame from early childhood to adolescence in the same model. Most prior research on this topic has focused on younger years or has examined these relations in a piecemeal manner across different developmental stages. Findings add to the current understanding of parenting effects by identifying which parenting behaviors can influence child EF and SR across development, how children’s EFs and SR influence parenting, and whether these effects are simply carried forward from younger years as early enduring effects or if there are additional, adolescence-specific associations between parenting behaviors and child EFs and SR that are not explained by lagged effects, early enduring effects, or late emerging effects. This test in particular is helpful in exploring the utility of parenting-based interventions, beyond early childhood years.

## Methods

### Sample Description

This study analyzed data from the Study of Early Child Care and Youth Development (SECCYD). SECCYD was a four-phase longitudinal study conducted from 1991 to 2008 by the National Institute of Child Health and Human Development (NICHD) to examine transactional linkages between specific parenting behaviors and child outcomes from early childhood to adolescence. Initial recruitment included a conditionally random sample of 3,015 mothers recruited from 24 designated hospitals across 10 U.S. cities. In order to be admitted into the study, mothers had to be at least 18 years of age, English speaking, and have no reported substance abuse history, and their newborns had to be healthy and free of disease and disability upon birth. Of these, 1,525 mothers were eligible and agreed to be interviewed.

The final Phase I (1991-1994) sample included 1,364 mothers; 65% had a high school degree or more, 11% had not completed high school, and 14% were single-parent families. The average family income was 3.6 times the poverty threshold. Infant demographics included 52% male and 76% non-Hispanic White, 13% African American, 6% Hispanic, and 5% Asian, Native American or other (Vandell & Gülseven, 2023). Phase I included assessments conducted from birth to 3 years. When children were 1 month of age, a home interview was conducted to collect demographic baseline data.

Phase II (1995-1999) included assessments conducted through 1^st^ grade (child age 7 years), Phase III (2000-2004) included assessments conducted through 6^th^ grade (child age 12 years), and Phase IV (2005-2007) included assessments conducted through 9^th^ grade (child age 15 years). There was 26% attrition across the waves (Phase II *N* = 1,226; Phase III *N* = 1,061; Phase IV *N* = 1,009). A detailed description of the data collection procedures and instruments can be found in the complete study manual (US Department of Health and Human Services, 2009). For the current analyses, we used assessments from early childhood (24, 36, and 54 months), middle childhood (Grades 1, 2, 3, 4, and 5), and adolescence (15 years). Some assessments from early childhood (i.e., 1, 6, and 15 month) and middle childhood (i.e., Kindergarten and Grade 6) were not included in current analyses because the same parenting constructs and child outcomes were not assessed at these waves.

### Measures

#### Parenting behaviors

Three parenting behaviors, namely autonomy support, supportive presence, and hostility were assessed using observational coding of the semi-structured Mother–Child Interaction Task, administered at multiple time points with task- and age-appropriate modifications. During early childhood, the mother-child interaction task involved mother and child engaging in free play with toys. In middle childhood, mother and child engaged in activity tasks (e.g., Etch-ASketch task), discussions regarding rules about what parents and children should do, and problem-solving tasks. During adolescence, mother and child engaged in discussions on topics of disagreement between them, selected by the adolescent from a list of possible topics. For details on the specific parent-child interaction tasks at each assessment, see Burchinal et al., 2014. Mother-child interactions were videotaped and coded at a central location using 7-point rating scales. All coders were blind to all information about the dyad. Inter-rater reliability was monitored throughout the coding period with intraclass correlations (ICCs) ranging from 0.84 to 0.91 (Wang et al., 2013). Similar to prior studies (e.g., Burchinal et al., 2014; Gülseven et a., 2021), which found that aggregating measurements across the developmental stages is helpful in creating more robust assessments of parenting constructs for that developmental period, multiple parenting assessments occurring within a single developmental stage (early childhood, middle childhood, adolescence) were standardized and averaged to create overall scores for the three parenting behaviors (autonomy support, supportive presence, and hostility) for each developmental period. This approach is further justified by the bivariate associations between the parenting variables, which were in the 0.30-0.50 range, across time points.

*Autonomy support* reflected mothers’ respect and support for child’s individuality, perspective, and motives (Bindman et al., 2015; Wang et al., 2013). High scores on this variable indicated that mothers allowed children choice and initiative, and acknowledged their opinions and actions. Low scores indicated that the mother was intrusive and controlling in her interactions with the child. Instead of treating the child as a partner in the interaction, mothers low on autonomy support were more likely to intrude harshly or coldly and deny the autonomy of the child without any acknowledgment of the intentions of the child (Wang et al., 2013). At the 24-month assessment, autonomy support was measured by reverse scoring the intrusiveness ratings. For all other assessment time points, autonomy support was assessed using the “respect for autonomy” variable coded on a scale from 0-7.

*Supportive presence* indexed the level of positive regard and emotional support from the mother to the child. We used observational coding scores of ‘positive regard’ to assess maternal positive response in early and middle childhood. In adolescence, observational coding scores of ‘valuing/ warmth’ were used. A mother high on this scale acknowledged the child’s accomplishments, encouraged the child’s efforts, and provided support and encouragement when the child was having difficulty. A mother low on this scale seldom provided supportive cues and was unavailable through passiveness and noninvolvement (Wang et al., 2013).

*Hostility* scores reflected mother’s expression of overt anger or discounting, rejecting, or blaming the child for mistakes (Bindman et al., 2015) and were derived from observational coding scores of maternal negative regard and hostility. Maternal negative regard captured the frequency and intensity of mother’s negative affect towards the child, such as anger, and rough physical actions. Maternal hostility captured negative emotionality, visible irritation with the child, providing negative feedback to the child, and rejecting or blaming of the child for mistakes (Bindman et al., 2015).

#### Child executive functions (EFs)

Child EFs were assessed using two core EFs – working memory and inhibitory control.

*Working memory* was assessed using Memory for Names (Sentences) scores on the Woodcock-Johnson-Revised (WJR) Cognitive subtest for working memory, as has been done by prior studies using this dataset (Hackman et al., 2015), and the Operation Span task (Turner & Engle, 1989). The early childhood working memory score was comprised of the Memory for Names score from the 5-year assessment. The middle childhood working memory score was comprised of the standardized average of the Memory for Names scores from the 7- and 9-year assessments. The adolescence working memory score was comprised of the Operation Span (O-Span) Task performance score from the age 15 assessment. In the Memory for Names subset of the WJ-R, a series of words, phrases, and sentences of increasing length and difficulty are presented orally and the child is asked to repeat exactly what was presented. The W score, a transformation of the Rasch ability scale centered on a value of 500 was used from this test. The W score is not standardized for relative rank and is an equal interval scale making it appropriate for examining growth over time. For the O-span task, the total span score, which represents the number of complete sets of correctly remembered letters, was used.

*Inhibitory Control* was measured using the Continuous Performance Task (CPT; Mirsky et al., 1991) and Stroop task (Gerstadt et al., 1994). In the CPT task, children were shown pictures of familiar items (e.g., a flower or a butterfly) on a computer screen and were asked to press a key only when they saw the target picture, which was a chair (Bindman et al., 2015). The total number of commission errors (i.e., button press responses to non-target stimuli) was reverse scored and used as a measure of inhibitory control, with higher scores indicating greater inhibitory control. For the Stroop task, children were shown a picture of the moon and stars or a picture of the sun and were instructed to say “day” when they saw the picture with the moon and stars and to say “night” when they saw the picture of the sun. The interference score on the Stroop task represented the percent of test items that were incorrectly answered, i.e., lower scores indicated less interference and greater inhibition skills. The variable was reverse scored such that higher scores indicated greater inhibitory control. For the early childhood inhibitory control assessment, the CPT and Stroop scores from the age 5 assessment were standardized and averaged. For the middle childhood inhibitory control assessment, we standardized and averaged the CPT assessments from child age 7 and 10 years. For the adolescence inhibitory control assessment, we used Stroop task score from the age 15 assessment.

*Self-regulation* (SR) was assessed using maternal reports on the 10-item self-control subscale of the Social Skills Rating System (SSRS; Gresham & Elliott, 1990). The scale measures caregiver (in this case mothers’) reports of their child’s ability to self-regulate their reactions in social contexts (e.g., “compromises in conflict situations,” “controls temper in conflict situations,” “responds appropriately to teasing”). Each item is rated on a 3-point response scale, ranging from 0 (never), 1 (sometimes), to 2 (very often). Mothers’ ratings of the self-control subscale were collected during early childhood (child age 5 years and 6 years), middle childhood (annually from child ages 7-12 years), and adolescence (child age 15 years). This measure had good internal consistency across the study period (alpha ranging from 0.78 – 0.83 (Vazsonyi & Jiskrova, 2018). Scores were standardized and averaged to create a composite SR score for each developmental period. As noted in the introduction, we are using the “self-control” dimension of self-regulation for the purposes of this study.

We chose not to include delay of gratification (assessed at child age 54 months) as an additional indicator of child SR because (a) there were no repeated assessments of this measure making it difficult to model change over time, and (b) combining this variable with the SSRS self-control (which is a self-report measure) may have introduced measurement bias given the different methods of measurement (behavioral vs. self-report).

#### Covariates

As specified in our preregistration, we controlled for child sex (male/female) and race-ethnicity (Non-Hispanic White/ Other racial-ethnic groups) as time-invariant covariates and family income-to-needs ratio and maternal depressive symptoms as time-varying covariates. Significant parenting-based differences have been observed based on child sex (Williams et al., 2002), family racial-ethnic background (Pachter et al., 2006), family SES (Roubinov & Boyce, 2017), and maternal depression (Lovejoy et al., 2000), making these important covariates to include in present analyses. Further, family SES and maternal depression can also influence child EFs and SR (Evans & English, 2002).

Child sex (male/female), race-ethnicity (White, African American, Hispanic, Asian, Native American, other), and other demographic information was obtained based on maternal reports at the child’s one-month interview. Due to the homogenous nature of the sample (76% non-Hispanic White), race-ethnicity was coded as a dummy variable (1= Other; 0 = Non-Hispanic White). For time-varying covariate of family income-to-needs ratio, we created composite scores for each developmental period by averaging assessments, based on maternal reports, for early childhood (1, 36, 54 months, and kindergarten assessments), middle childhood (Grade 1, 3, 4, 5, 6) and adolescence (age 15 assessment). Maternal depressive symptoms were assessed using the Center for Epidemiological Studies Depression (CES-D) scale. The CES-D scale included 20 depression symptoms for which mothers were asked to report the frequency with which they experienced those symptoms over the past week. Maternal depressive symptom scores were calculated based on maternal reports for early childhood (combining 1, 15, 24, 36, and 54-month assessments), middle childhood (combining Grade 1, 3, and 5 assessments) and adolescence (age 15 assessment).

### Statistical Analysis

We examined transactional linkages between parenting behaviors and child outcomes across early childhood (24 , 36, and 54 months), middle childhood (Grades 1, 2, 3, 4, and 5), and adolescence (15 years) using random intercept crosslagged panel models (RI-CLPMs) in M*plus* 8.0 (Muthén & Muthén, 1998-2017). Some assessments from early childhood (i.e., 1, 6, and 15 month) and middle childhood (i.e., Kindergarten and Grade 6) were not included in current analyses because the same parenting constructs and child outcomes were not assessed at these waves. RI-CLPM is an advanced version of standard cross-lagged panel modeling (CLPM) that provides a more-stringent test of potential causal relations between variables and is appropriate for examining transactional over time linkages between variables of interest (Usami, 2021).

Whereas standard cross-lagged panel modeling (CLPM) confounds between- and within-subject associations, RICLPM isolates changes at a within-subject level as predictors of subsequent within-subject changes. Like standard CLPM, RI-CLPM includes stability pathways for outcomes over time and cross-lagged paths to describe the transactional associations between variables. These stability and cross-lagged paths represent how variation in levels of a variable within a subject predicts change in those and other variables within the same subject over time. Correlations among the latent between-subjects level variables represent whether subjects (e.g., mother-child dyads) who are higher (or lower) overall on one construct across waves (e.g., maternal autonomy support) as compared to other families are also higher (or lower) overall on another construct (e.g., child working memory). See Hamaker et al. (2015) and Lucas (2023) for a more technical explanation. We chose RICLPM over other trajectory modeling techniques (e.g., ALTSR) because we were more interested in the temporal associations between the two variables over time, rather than predicting individual changes over time.

**Figure 1:**
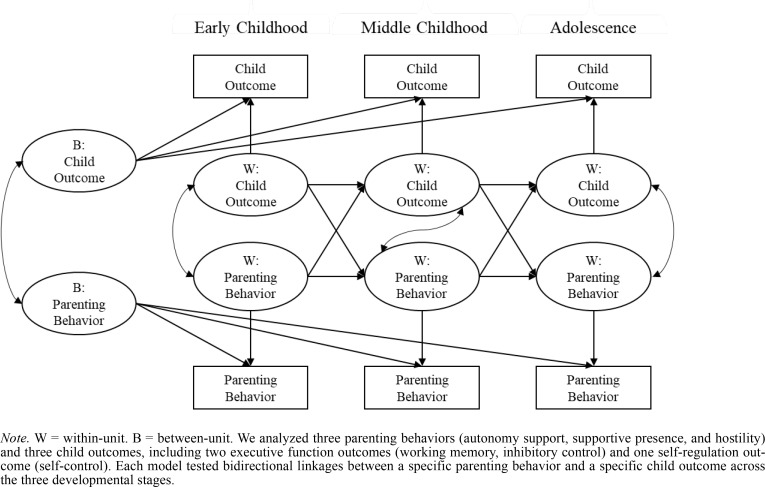
Path Diagram for a Random Intercept Cross-Lagged Panel Model

A total of nine RI-CLPMs were tested, for each parenting behavior-child outcome combination as conceptualized in Figure 1. We analyzed three parenting behaviors (autonomy support, supportive presence, and hostility) and three child outcomes, including two executive function outcomes (working memory, inhibitory control) and one self-regulation outcome (self-control). Each model tested bidirectional linkages between the specific parenting behavior and specific child outcome across the three developmental stages. Including multiple parenting or child variables in the RICLPMs increases the number of free parameters and produces results that are difficult to interpret. To maintain model parsimony and interpretability of results, we utilized nine separate RI-CLPMs to examine the specific transactional associations between each parenting behavior and each child outcome, separately.

We performed analyses with and without adjusting for covariates. Adjusted models included child gender and ethnicity as time-invariant covariates and family income-to-needs ratio and maternal depression as time-varying covariates. We fit the RI-CLPMs using maximum likelihood estimation with robust standard errors, which is robust against deviations from the normality assumption. Model fit was determined based on traditional indices, with good fit indicated by RMSEA values less than .06, CFI values greater than .95, and SRMR values less than .08 (Hu & Bentler, 1999).

Multiple imputation (MI) in M*plus* was used to impute missing values on all study variables. Rates of missing data ranged from 11-23% in early childhood, 17-21% in middle childhood, and 27-34% in adolescence. MI is an acceptable solution for dealing with missing data regardless of whether the data are MAR (Rubin, 1996; Collins et al., 2001) and has been demonstrated to produce similar results across data that are missing completely at random, MAR, and not missing at random (Gadbury et al., 2003). The MI procedure generated 20 complete data sets using all available self-report measures as predictors of missing values. Analyses were conducted for each predictor of class membership across each of the 20 imputed data sets and pooled estimates are reported in the results.

For late emerging effects, we tested direct effects of the three parenting behaviors in early childhood on adolescent outcomes by re-specifying the adjusted RI-CLPMs to include the direct paths from early childhood parenting to adolescent outcomes of working memory, inhibitory control, and SR. Since the model accounted for stability and crosslagged effects, these pathways represent effects of early parenting that manifest in later years.

To test for unique adolescent-specific effects, we fixed the covariance between parenting at age 15 and child outcome at age 15 to zero, and compared nested models with and without the constraint using a likelihood ratio test. If there was a significant drop in model fit, and the residual covariance was significant, that indicated that there was significant covariance between parenting and child outcome at age 15 that was not explained by early enduring effects, lagged effects or late emerging effects. Indirect effects hypotheses were tested using time-ordered mediation modeling (Cole & Maxwell, 2003; MacKinnon, 2008). Indirect effects of early parenting on age 15 child self-regulation were tested for each of the three parenting behaviors, with two mediators (inhibitory control and working memory) in separate models (total indirect effects tested = 6). Bootstrapped standard errors were used for the asymptotically distributed multiplicative indirect terms, along with bias corrected confidence intervals (Preacher & Hayes, 2008)

## Results

**Figure 2:**
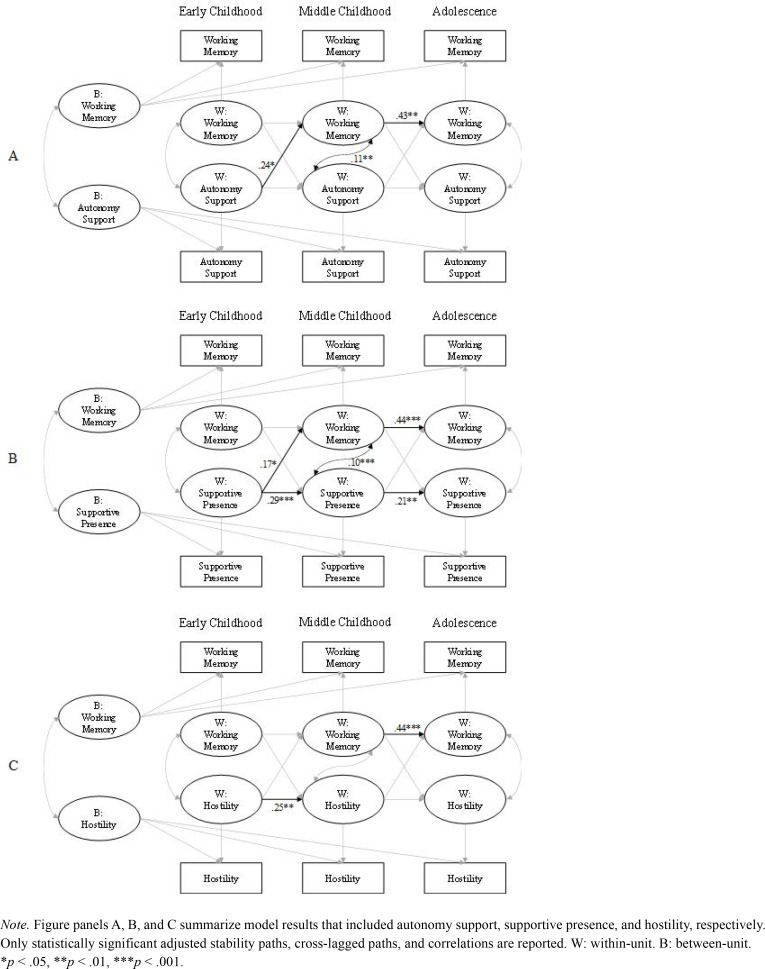
Results of Random Intercept Cross-Lagged Panel Models (Adjusted) Linking Each Parenting Behavior and Working Memory.

**Figure 3:**
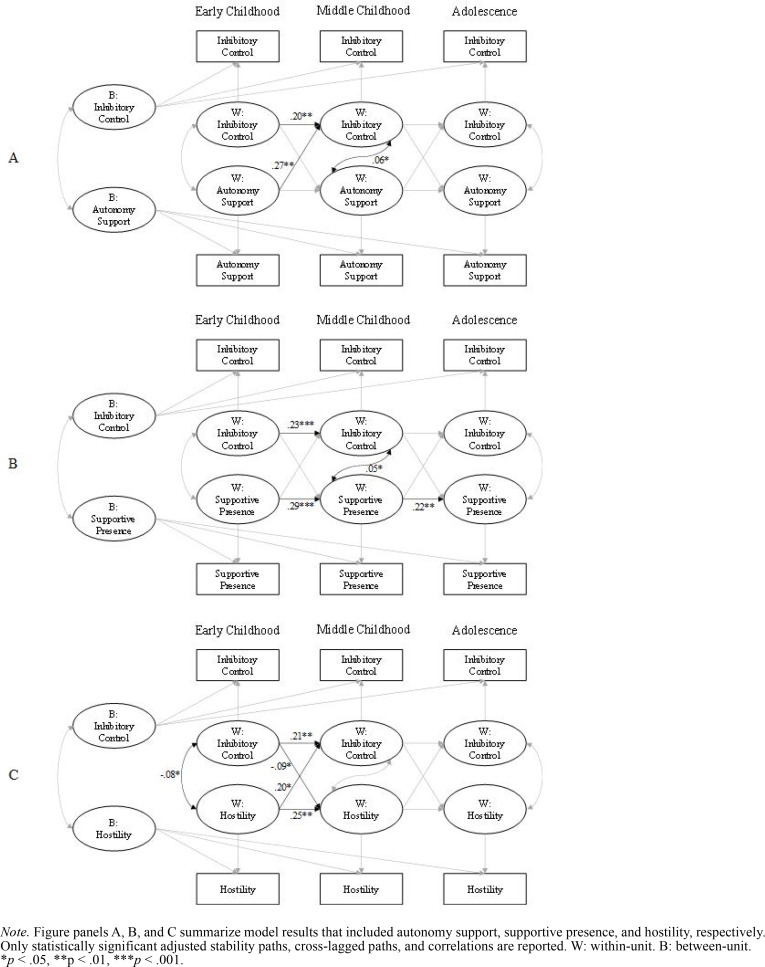
Results of Random Intercept Cross-Lagged Panel Models (Adjusted) Linking Each Parenting Behavior and Inhibitory Control.

**Figure 4:**
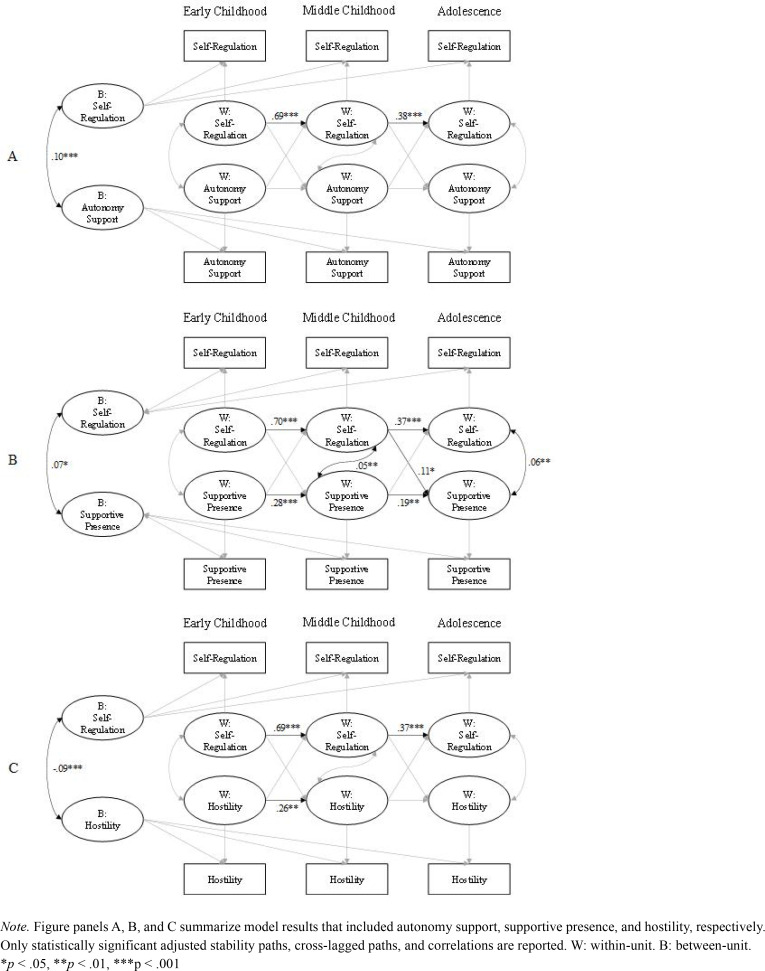
Results of Random Intercept Cross-Lagged Panel Models (Adjusted) Linking Each Parenting Behavior and Self-Regulation.

We conducted preliminary analyses to determine how much of the variability in parenting behaviors and child outcomes was due to between-subject differences versus within-subject variations across developmental periods. We calculated intraclass correlation coefficients (ICCs) for each outcome to describe the percent of variation occurring between- versus within-subjects. ICCs for parenting behaviors were .66 for autonomy support, .53 for hostility, and .69 for supportive presence, indicating that 53-69% of the variation in parenting variables occurred betweensubjects (31-47% of variation occurred within-subjects over time). ICCs for child outcomes were .46 for working memory, .25 for inhibitory control, and .78 for SR. These results indicated that there was sufficient within-subject variation to estimate RI-CLPMs. The correlations between parenting behaviors and child outcomes across the three developmental periods are reported in Table 1. Results from the nine RI-CLPMs are described below, organized by three child outcomes (also see Figures 2-4 and Tables 2-9).

**Table 1:** Correlations among Parenting Behaviors and Child Outcomes across Developmental Periods.

Variable		Dev. Period	*M* (*SD*)	1	2	3	4	5	6	7	8	9	10	11	12	13	14	15	16	17
1	Self-regulation	EC	-.02 (.72)																	
2		MC	-.01 (.89)	.67																
3		A	-.02 (.78)	.40	.54															
4	Inhibitory control	EC	-.02 (.87)	.17	.10	.05														
5		MC	-.01 (.89)	.18	.11	.13	.26													
6		A	.00 (1.00)	.05	.02	.05	.02	.07												
7	Working memory	EC	.00 (1.00)	.08	.06	.07	.04	.08	.03											
8		MC	-.01 (.95)	.28	.24	.20	.15	.21	.09	.17										
9		A	.00 (1.00)	.16	.14	.15	.12	.13	.08	.10	.45									
10	Autonomy support	EC	-.02 (.80)	.32	.31	.31	.23	.30	.04	.08	.33	.17								
11		MC	-.02 (.81)	.33	.33	.31	.22	.24	.03	.06	.37	.21	.53							
12		A	.00 (1.00)	.21	.23	.30	.13	.13	.00	.07	.15	.03	.33	.36						
13	Hostility	EC	.01 (.79)	-.27	-.24	-.27	-.24	-.26	-.04	-.04	-.25	-.14	-.68	-.44	-.25					
14		MC	.01 (.80)	-.27	-.28	-.29	-.21	-.17	-.03	-.06	-.20	-.14	-.38	-.64	-.28	.46				
15		A	.00 (1.00)	-.14	-.14	-.19	-.07	-.09	-.03	-.02	-.04	-.02	-.23	-.22	-.48	.23				
16	Supportive presence	EC	-.01 (.79)	.27	.26	.26	.17	.23	.03	.08	.32	.15	.65	.46	.28	-.61	-.32	-.20		
17		MC	-.01 (.83)	.34	.35	.32	.21	.24	.03	.06	.36	.19	.53	.81	.37	-.45	-.64	-.22	.56	
18		A	.00 (.81)	.22	.27	.29	.14	.10	.00	.06	.19	.04	.31	.34	.60	-.23	-.23	-.37	.30	.40

*Note. M* = mean, *SD* = standard deviation, EC = early childhood, MC = middle childhood, A = adolescence. Pairwise sample sizes ranged from 822 to 1,227. Correlations > .10 were statistically significant at *p* < .05.

### Parenting Behaviors and Child Working Memory

The three RI-CLPMs for each specific parenting behavior and child working memory fit the data well. See Tables 2 to 4 for fit statistics and complete parameter estimates for models involving autonomy support, supportive presence, and hostility, respectively. See Figure 2 for statistically significant covariate-adjusted parameter estimates. We emphasize covariate-adjusted stability and cross-lagged pathways in the results described below and report both the unadjusted and adjusted results in the tables.

**Table 2:** Results of Random Intercept Cross-Lagged Panel Models Linking Autonomy Support and Working Memory.

Model and Parameters	β (*SE*)	*p*	β (*SE*)	*p*	β (*SE*)	*p*
Unadjusted model						
Stability path	Early to Middle Childhood		Middle Childhood to Adolescence			
Autonomy support	0.27*** (0.06)	<.001	0.07 (0.09)	.402		
Working memory	0.12 (0.07)	.100	0.43*** (0.06)	<.001		
Cross-lagged path						
Autonomy support --> Working memory	0.53*** (0.08)	<.001	0.05 (0.07)	.450		
Working memory --> Autonomy support	-0.01 (0.03)	.852	0.10 (0.06)	.083		
Correlation/Correlated change	Early Childhood		Middle Childhood		Adolescence	
Autonomy support <--> Working memory	0.00 (0.04)	.920	0.19*** (0.03)	<.001	-0.07* (0.03)	.032
Between-person covariance	Across Waves					
Autonomy support <--> Working memory	0.06 (0.03)	.074				

Adjusted model						
Stability path	Early to Middle Childhood		Middle Childhood to Adolescence			
Autonomy support	0.03 (0.08)	.696	0.05 (0.09)	.565		
Working memory	0.07 (0.06)	.237	0.43*** (0.06)	<.001		
Cross-lagged path						
Autonomy support --> Working memory	0.24* (0.10)	.021	0.06 (0.07)	.370		
Working memory --> Autonomy support	-0.04 (0.03)	.227	0.00 (0.05)	.964		
Correlation/Correlated change	Early Childhood		Middle Childhood		Adolescence	
Autonomy support <--> Working memory	-0.03 (0.04)	.485	0.11** (0.03)	.001	-0.06 (0.03)	.051
Between-person covariance	Across Waves					
Autonomy support <--> Working memory	0.05 (0.03)	.067				

*Note.* RI-CLPM = random intercept cross-lagged panel model. Model fit was acceptable: RMSEA = .067, CFI = 1.000, SRMR = .003. 95% CI for parameter estimates can be derived: β ± 1.96 × *SE.*

* *p* < .05,

** *p* < .01,

*** *p* < .001.

For the adjusted stability pathways, the path for working memory was significant from early to middle childhood but not from middle childhood to adolescence (Figure 2; Tables 2, 3, and 4). Stability paths for autonomy support were not significant for either time lag (Table 2). Both stability paths for supportive presence, from early to middle childhood and from middle childhood to adolescence, were significant (Table 3). The stability path for hostility was significant from early to middle childhood but not from middle childhood to adolescence (Table 4).

**Table 3:** Results of Random Intercept Cross-Lagged Panel Models Linking Supportive Presence and Working Memory.

Model and Parameters	β (*SE*)	*p*	β (*SE*)	*p*	β (*SE*)	*p*
Unadjusted model						
Stability path	Early to Middle Childhood		Middle Childhood to Adolescence			
Supportive presence	0.51*** (0.05)	<.001	0.23*** (0.06)	<.001		
Working memory	0.11 (0.07)	.123	0.44*** (0.05)	<.001		
Cross-lagged path						
Supportive presence --> Working memory	0.42*** (0.06)	<.001	0.04 (0.05)	.515		
Working memory --> Supportive presence	0.01 (0.03)	.794	0.08 (0.04)	.076		
Correlation/Correlated change	Early Childhood		Middle Childhood		Adolescence	
Supportive presence <--> Working memory	0.03 (0.04)	.527	0.16*** (0.03)	<.001	-0.05* (0.03)	.040
Between-person covariance	Across Waves					
Supportive presence <--> Working memory	0.04 (0.03)	.182				

Adjusted model						
Stability path	Early to Middle Childhood		Middle Childhood to Adolescence			
Supportive presence	0.29*** (0.07)	<.001	0.21** (0.06)	.001		
Working memory	0.07 (0.06)	.221	0.44*** (0.05)	<.001		
Cross-lagged path						
Supportive presence --> Working memory	0.17* (0.08)	.028	0.03 (0.06)	.618		
Working memory --> Supportive presence	-0.03 (0.02)	.266	0.04 (0.04)	.411		
Correlation/Correlated change	Early Childhood		Middle Childhood		Adolescence	
Supportive presence <--> Working memory	-0.01 (0.04)	.738	0.10*** (0.03)	<.001	-0.04 (0.03)	.086
Between-person covariance	Across Waves					
Supportive presence <--> Working memory	0.04 (0.03)	.103				

*Note.* RI-CLPM = random intercept cross-lagged panel model. Model fit was acceptable: RMSEA = .010, CFI = .999, SRMR = .003. 95% CI for parameter estimates can be derived: β ± 1.96 × *SE.*

* *p* < .05,

** *p* < .01,

*** *p* < .001.

**Table 4:** Results of Random Intercept Cross-Lagged Panel Models Linking Hostility and Working Memory.

Model and Parameters	β (*SE*)	*p*	β (*SE*)	*p*	β (*SE*)	*p*
Unadjusted model						
Stability path	Early to Middle Childhood		Middle Childhood to Adolescence			
Hostility	0.30*** (0.08)	<.001	0.05 (0.08)	.512		
Working memory	0.13 (0.07)	.075	0.44*** (0.05)	<.001		
Cross-lagged path						
Hostility --> Working memory	-0.39*** (0.08)	<.001	-0.08 (0.06)	.215		
Working memory --> Hostility	-0.02 (0.04)	.580	-0.03 (0.05)	.549		
Correlation/Correlated change	Early Childhood		Middle Childhood		Adolescence	
Hostility <--> Working memory	0.00 (0.04)	.900	-0.08** (0.03)	.002	0.01 (0.04)	.765
Between-person covariance	Across Waves					
Hostility <--> Working memory	-0.03 (0.03)	.384				

Adjusted model						
Stability path	Early to Middle Childhood		Middle Childhood to Adolescence			
Hostility	0.25** (0.08)	.002	0.09 (0.08)	.251		
Working memory	0.08 (0.06)	.176	0.44*** (0.05)	<.001		
Cross-lagged path						
Hostility --> Working memory	-0.16 (0.08)	.057	-0.09 (0.06)	.155		
Working memory --> Hostility	-0.01 (0.04)	.822	0.04 (0.05)	.491		
Correlation/Correlated change	Early Childhood		Middle Childhood		Adolescence	
Hostility <--> Working memory	0.03 (0.04)	.458	-0.04 (0.03)	.119	0.01 (0.04)	.862
Between-person covariance	Across Waves					
Hostility <--> Working memory	-0.02 (0.03)	.396				

*Note.* RI-CLPM = random intercept cross-lagged panel model. Model fit was acceptable: RMSEA = .015, CFI = .999, SRMR = 005. 95% CI for parameter estimates can be derived: β ± 1.96 × *SE.*

* *p* < .05,

** *p* < .01,

*** *p* < .001.

Regarding the cross-lagged paths, greater autonomy support and supportive presence in early childhood was significantly associated with greater child working memory in middle childhood (Tables 3 and 4, respectively). No other adjusted cross-lagged paths were statistically significant. There was no evidence of evocative child working memory effects on parenting at any of the time points in the adjusted or unadjusted models (Tables 2-4).

### Parenting Behaviors and Child Inhibitory Control

The RI-CLPMs for the three parenting behaviors and child inhibitory control fit the data well (see Tables 5 to 7 for fit statistics and complete parameter estimates, and Figure 3 for statistically significant covariate-adjusted parameter estimates). Similar to working memory, the adjusted stability path for inhibitory control was significant from early to middle childhood but not from middle childhood to adolescence (Figure 3; Tables 5, 6, and 7). The pattern of significance for stability paths for autonomy support, supportive presence, and hostility remained the same as in the first set of models with child working memory.

**Table 5:** Results of Random Intercept Cross-Lagged Panel Models Linking Autonomy Support and Inhibitory Control.

Model and Parameters	β (*SE*)	*p*	β (*SE*)	*p*	β (*SE*)	*p*
Unadjusted model						
Stability path	Early to Middle Childhood		Middle Childhood to Adolescence			
Autonomy support	0.24*** (0.07)	<.001	0.11 (0.08)	.173		
Inhibitory control	0.20** (0.06)	.001	0.05 (0.07)	.438		
Cross-lagged path						
Autonomy support --> Inhibitory control	0.33** (0.10)	.001	-0.06 (0.08)	.469		
Inhibitory control --> Autonomy support	0.10** (0.04)	.009	0.03 (0.06)	.633		
Correlation/Correlated change	Early Childhood		Middle Childhood		Adolescence	
Autonomy support <--> Inhibitory control	0.10** (0.03)	.003	0.07* (0.03)	.013	-0.05 (0.04)	.192
Between-person covariance	Across Waves					
Autonomy support <--> Inhibitory control	0.07* (0.03)	.018				

Adjusted model						
Stability path	Early to Middle Childhood		Middle Childhood to Adolescence			
Autonomy support	0.05 (0.08)	.546	0.07 (0.09)	.452		
Inhibitory control	0.20** (0.06)	.001	0.06 (0.07)	.377		
Cross-lagged path						
Autonomy support --> Inhibitory control	0.27** (0.10)	.008	-0.03 (0.08)	.673		
Inhibitory control --> Autonomy support	0.06 (0.04)	.152	0.01 (0.06)	.822		
Correlation/Correlated change	Early Childhood		Middle Childhood		Adolescence	
Autonomy support <--> Inhibitory control	0.05 (0.03)	.087	0.06* (0.03)	.027	-0.01 (0.03)	.705
Between-person covariance	Across Waves					
Autonomy support <--> Inhibitory control	0.03 (0.02)	.151				

*Note.* RI-CLPM = random intercept cross-lagged panel model. Model fit was acceptable: RMSEA = .067, CFI = .991, SRMR = .015. 95% CI for parameter estimates can be derived: β ± 1.96 × *SE.*

* *p* < .05,

** *p* < .01,

*** *p* < .001.

**Table 6:** Results of Random Intercept Cross-Lagged Panel Models Linking Supportive Presence and Inhibitory Control.

Model and Parameters	β (*SE*)	*p*	β (*SE*)	*p*	β (*SE*)	*p*
Unadjusted model						
Stability path	Early to Middle Childhood		Middle Childhood to Adolescence			
Supportive presence	0.49*** (0.05)	<.001	0.26*** (0.06)	<.001		
Inhibitory control	0.24*** (0.06)	<.001	0.06 (0.07)	.367		
Cross-lagged path						
Supportive presence --> Inhibitory control	0.18* (0.07)	.014	-0.07 (0.07)	.286		
Inhibitory control --> Supportive presence	0.09** (0.03)	.006	-0.04 (0.05)	.433		
Correlation/Correlated change	Early Childhood		Middle Childhood		Adolescence	
Supportive presence <--> Inhibitory control	0.06 (0.03)	.060	0.06** (0.02)	.008	-0.05 (0.03)	.172
Between-person covariance	Across Waves					
Supportive presence <--> Inhibitory control	0.06 (0.03)	.053				

Adjusted model						
Stability path	Early to Middle Childhood		Middle Childhood to Adolescence			
Supportive presence	0.29*** (0.07)	<.001	0.22** (0.06)	.001		
Inhibitory control	0.23*** (0.06)	<.001	0.07 (0.07)	.311		
Cross-lagged path						
Supportive presence --> Inhibitory						
control	0.11 (0.08)	.152	-0.08 (0.07)	.263		
Inhibitory control --> Supportive presence	0.05 (0.03)	.188	-0.04 (0.05)	.394		
Correlation/Correlated change	Early Childhood		Middle Childhood		Adolescence	
Supportive presence <--> Inhibitory control	0.01 (0.03)	.705	0.05* (0.02)	.034	-0.02 (0.03)	.545
Between-person covariance	Across Waves					
Supportive presence <--> Inhibitory control	0.04 (0.03)	.121				

*Note.* RI-CLPM = random intercept cross-lagged panel model. Model fit was acceptable: RMSEA = .044, CFI = .996, SRMR = .009. 95% CI for parameter estimates can be derived: β ± 1.96 × *SE.*

* *p* < .05,

** *p* < .01,

*** *p* < .001.

**Table 7:** Results of Random Intercept Cross-Lagged Panel Models Linking Hostility and Inhibitory Control.

Model and Parameters	β (*SE*)	*p*	β (*SE*)	*p*	β (*SE*)	*p*
Unadjusted model						
Stability path	Early to Middle Childhood		Middle Childhood to Adolescence			
Hostility	0.29*** (0.07)	<.001	0.07 (0.08)	.410		
Inhibitory control	0.22*** (0.06)	<.001	0.06 (0.07)	.381		
Cross-lagged path						
Hostility --> Inhibitory control	-0.24** (0.08)	.004	0.03 (0.07)	.646		
Inhibitory control --> Hostility	-0.10** (0.03)	.004	-0.03 (0.07)	.717		
Correlation/Correlated change	Early Childhood		Middle Childhood		Adolescence	
Hostility <--> Inhibitory control	-0.11** (0.04)	.003	-0.02 (0.03)	.387	0.02 (0.04)	.715
Between-person covariance	Across Waves					
Hostility <--> Inhibitory control	-0.05 (0.03)	.123				

Adjusted model						
Stability path	Early to Middle Childhood		Middle Childhood to Adolescence			
Hostility	0.25** (0.08)	.001	0.10 (0.08)	.197		
Inhibitory control	0.21** (0.06)	.001	0.06 (0.07)	.329		
Cross-lagged path						
Hostility --> Inhibitory control	-0.20* (0.08)	.013	0.00 (0.07)	.967		
Inhibitory control --> Hostility	-0.09* (0.03)	.010	-0.01 (0.07)	.851		
Correlation/Correlated change	Early Childhood		Middle Childhood		Adolescence	
Hostility <--> Inhibitory control	-0.08* (0.04)	.023	-0.02 (0.02)	.334	0.00 (0.04)	.921
Between-person covariance	Across Waves					
Hostility <--> Inhibitory control	-0.03 (0.03)	.388				

*Note.* RI-CLPM = random intercept cross-lagged panel model. Model fit was acceptable: RMSEA = .027, CFI = .997, SRMR = .007. 95% CI for parameter estimates can be derived: β ± 1.96 × *SE.*

* *p* < .05,

** *p* < .01,

*** *p* < .001.

For the cross-lagged paths, greater autonomy support in early childhood was significantly associated with greater child inhibitory control in middle childhood (Table 5). Greater hostility in early childhood was significantly associated with lower inhibitory control in middle childhood (Table 7). In terms of child effects, stronger inhibitory control in early childhood was significantly associated with less maternal hostility in middle childhood (Table 7). No other adjusted cross-lagged paths were statistically significant.

### Parenting Behaviors and Child Self-Regulation

The RI-CLPMs for the three parenting behaviors and child self-regulation also fit the data well. Tables 8 to 10 report fit statistics and parameter estimates, and Figure 4 reports statistically significant covariate-adjusted parameter estimates. The adjusted stability paths for self-regulation were significant from early to middle childhood and from middle childhood to adolescence (Figure 4; Tables 8, 9, and 10). The pattern of significance for stability paths for autonomy support, supportive presence, and hostility remained the same as prior models.

**Table 8:** Results of Random Intercept Cross-Lagged Panel Models Linking Autonomy Support and Self-Regulation.

Model and Parameters	β (*SE*)	*p*	β (*SE*)	*p*	β (*SE*)	*p*
Unadjusted model						
Stability path	Early to Middle Childhood		Middle Childhood to Adolescence			
Autonomy support	0.22** (0.07)	.002	0.09 (0.09)	.303		
Self-regulation	0.77*** (0.04)	<.001	0.39*** (0.04)	<.001		
Cross-lagged path						
Autonomy support --> Self-regulation	0.13* (0.05)	.011	0.04 (0.05)	.453		
Self-regulation --> Autonomy support	0.12* (0.05)	.030	0.07 (0.05)	.161		
Correlation/Correlated change	Early Childhood		Middle Childhood		Adolescence	
Autonomy support <--> Self-regulation	0.05 (0.03)	.103	0.04** (0.02)	.008	0.06** (0.02)	.008
Between-person covariance	Across Waves					
Autonomy support <--> Self-regulation	0.15*** (0.03)	<.001				

Adjusted model						
Stability path	Early to Middle Childhood		Middle Childhood to Adolescence			
Autonomy support	0.04 (0.09)	.668	0.06 (0.09)	.495		
Self-regulation	0.69*** (0.05)	<.001	0.38*** (0.04)	<.001		
Cross-lagged path						
Autonomy support --> Self-regulation	0.02 (0.06)	.685	0.04 (0.05)	.482		
Self-regulation --> Autonomy support	-0.01 (0.06)	.909	0.04 (0.06)	.511		
Correlation/Correlated change	Early Childhood		Middle Childhood		Adolescence	
Autonomy support <--> Self-regulation	-0.01 (0.02)	.791	0.02 (0.02)	.163	0.07** (0.02)	.001
Between-person covariance	Across Waves					
Autonomy support <--> Self-regulation	0.10*** (0.02)	<.001				

*Note.* RI-CLPM = random intercept cross-lagged panel model. Model fit was acceptable: RMSEA = .031, CFI = .999, SRMR = .015. 95% CI for parameter estimates can be derived: β ± 1.96 × *SE.*

* *p* < .05,

** *p* < .01,

*** *p* < .001.

**Table 9:** Results of Random Intercept Cross-Lagged Panel Models Linking Supportive Presence and Self-regulation.

Model and Parameters	β (*SE*)	*p*	β (*SE*)	*p*	β (*SE*)	*p*
Unadjusted model						
Stability path	Early to Middle Childhood		Middle Childhood to Adolescence			
Supportive presence	0.47*** (0.05)	<.001	0.23*** (0.06)	<.001		
Self-regulation	0.78*** (0.04)	<.001	0.38*** (0.04)	<.001		
Cross-lagged path						
Supportive presence --> Self-regulation	0.10* (0.04)	.015	0.11* (0.05)	.021		
Self-regulation --> Supportive presence	0.22*** (0.05)	<.001	0.10* (0.04)	.022		
Correlation/Correlated change	Early Childhood		Middle Childhood		Adolescence	
Supportive presence <--> Self-regulation	0.08 (0.04)	.051	0.06*** (0.02)	<.001	0.04* (0.02)	.038
Between-person covariance	Across Waves					
Supportive presence <--> Self-regulation	0.09* (0.04)	.015				

Adjusted model						
Stability path	Early to Middle Childhood		Middle Childhood to Adolescence			
Supportive presence	0.28*** (0.07)	<.001	0.19** (0.06)	.003		
Self-regulation	0.70*** (0.04)	<.001	0.37*** (0.04)	<.001		
Cross-lagged path						
Supportive presence --> Self-regulation	0.02 (0.05)	.708	0.09 (0.05)	.079		
Self-regulation --> Supportive presence	0.10 (0.06)	.096	0.11* (0.04)	.012		
Correlation/Correlated change	Early Childhood		Middle Childhood		Adolescence	
Supportive presence <--> Self-regulation	0.01 (0.03)	.815	0.05** (0.02)	.006	0.06** (0.02)	.003
Between-person covariance	Across Waves					
Supportive presence <--> Self-regulation	0.07* (0.03)	.019				

*Note.* RI-CLPM = random intercept cross-lagged panel model. Model fit was acceptable: RMSEA = .028, CFI = .999, SRMR = .006. 95% CI for parameter estimates can be derived: β ± 1.96 × *SE.*

* *p* < .05,

** *p* < .01,

*** *p* < .001.

**Table 10:** Results of Random Intercept Cross-Lagged Panel Models Linking Hostility and Self-regulation.

Model and Parameters	β (*SE*)	*p*	β (*SE*)	*p*	β (*SE*)	*p*
Unadjusted model						
Stability path	Early to Middle Childhood		Middle Childhood to Adolescence			
Hostility	0.28*** (0.08)	<.001	0.06 (0.09)	.472		
Self-regulation	0.79*** (0.04)	<.001	0.40*** (0.04)	<.001		
Cross-lagged path						
Hostility --> Self-regulation	-0.04 (0.04)	.422	-0.04 (0.05)	.446		
Self-regulation --> Hostility	-0.07 (0.06)	.235	0.02 (0.06)	.784		
Correlation/Correlated change	Early Childhood		Middle Childhood		Adolescence	
Hostility <--> Self-regulation	-0.02 (0.03)	.479	-0.04* (0.02)	.025	-0.02 (0.03)	.521
Between-person covariance	Across Waves					
Hostility <--> Self-regulation	-0.14*** (0.03)	<.001				

Adjusted model						
Stability path	Early to Middle Childhood		Middle Childhood to Adolescence			
Hostility	0.26** (0.08)	.001	0.11 (0.08)	.177		
Self-regulation	0.69*** (0.05)	<.001	0.37*** (0.04)	<.001		
Cross-lagged path						
Hostility --> Self-regulation	0.04 (0.05)	.382	-0.07 (0.05)	.159		
Self-regulation --> Hostility	-0.04 (0.06)	.580	0.04 (0.06)	.467		
Correlation/Correlated change	Early Childhood		Middle Childhood		Adolescence	
Hostility <--> Self-regulation	0.01 (0.03)	.637	-0.03 (0.02)	.078	-0.03 (0.03)	.250
Between-person covariance	Across Waves					
Hostility <--> Self-regulation	-0.09*** (0.03)	<.001				

*Note.* RI-CLPM = random intercept cross-lagged panel model. Model fit was acceptable: RMSEA = .044, CFI = .998, SRMR = .009. 95% CI for parameter estimates can be derived: β ± 1.96 × *SE.*

* *p* < .05,

** *p* < .01,

*** *p* < .001.

For cross-lagged paths, none of the parenting effects on child SR were significant. In case of child effects, higher child SR in middle childhood was significantly associated with greater supportive presence in adolescence (Table 9). No other adjusted cross-lagged paths were statistically significant.

### Late-Emerging Effects of Early Parenting on Adolescent Outcomes

We found no late-emerging effects of early parenting on any of the adolescent outcomes (*p*’s > .317). That is, accounting for the lagged effects, parenting in early childhood did not have any significant direct associations with child EF and SR outcomes in adolescence.

### Indirect Effects of Parenting on Child Self-Regulation

We also tested indirect effects of early parenting on child SR in adolescence, mediated by child EFs in middle childhood. These indirect effects were tested using extended three-variable specifications of the adjusted RI-CLPMs, with each of the three parenting behaviors and each of the two mediators (child working memory and inhibitory control) in separate models (total number of indirect effects tested = 6). Bootstrapped standard errors were used for the asymptotically distributed multiplicative indirect terms, along with bias corrected confidence intervals (Preacher & Hayes, 2008). We found no statistically significant indirect effects (p’s > .210). We also found no significant indirect effects of early parenting on child SR in middle childhood, channeled through child EFs in middle childhood (*p*’s > .585).

### Adolescence-Specific Associations between Parenting and Child Outcomes

Our final two-variable specifications of the adjusted RICLPMs tested for any residual covariances between parenting and child outcomes in adolescence. Specifically, in each of the nine RI-CLPMs, we fixed the covariance between parenting at age 15 and child outcome at age 15 to zero and compared nested models with and without the constraint using a likelihood ratio test. When there was a significant drop in model fit, and the residual covariance was significant, it indicated that there was significant covariance between parenting and child outcome in adolescence that was not explained by early enduring effects, lagged effects, or late emerging effects. We found significant residual covariances in adolescence between autonomy support and child SR (*β* = .07, *p* = .001), and between supportive presence and child SR (*β* = .06, *p* = .003).

## Discussion

The purpose of this study was to examine the transactional linkages between three specific parenting behaviors and child EFs and SR across the developmental periods of early childhood, middle childhood, and adolescence. In addition to modeling the stability and lagged pathways in RI-CLPMs, we tested for early enduring effects, late emerging effects, and residual covariance between parenting and child outcomes in adolescence accounting for these effects. We also evaluated potential indirect effects of early parenting on adolescent SR as mediated by child EFs. Overall, we found evidence for significant parenting effects on change in child EFs (but not child SR) from early to middle childhood, and not from middle childhood to adolescence. For child effects on parenting behaviors, we found evidence for child inhibitory control impacting parenting behavior change from early to middle childhood and child SR impacting parenting behavior change from middle childhood to adolescence. There was no significant indirect effect of parenting on child SR mediated by child EFs. We found significant residual covariance between parenting and child SR in adolescence which was not accounted for by early enduring, lagged, or late emerging effects, suggesting that there are associations between parenting and child SR that are unique to the adolescent period. We did not find any adolescent-specific effects of parenting on child EF. Below, we discuss our findings in terms of the main themes that emerged from the analyses.

### Parenting Effects on Child Outcomes: Relative Magnitude and Timing of Effects

As hypothesized, autonomy support had the strongest effects on child EFs as compared to supportive presence and hostility. Autonomy supportive parenting in early childhood was predictive of stronger child working memory and inhibitory control in middle childhood, controlling for stability pathways. Of the other parenting behaviors, supportive presence in early childhood was a significant predictor of child working memory, and hostility was a significant predictor of child inhibitory control in middle childhood, controlling for stability pathways. Overall, the effect of autonomy support was broader as compared to supportive presence and hostility, evidenced in its positive associations with both child working memory and inhibitory control. This may be because mothers who engage in autonomy supportive parenting provide children with the independence and scaffolding necessary to develop a sense of self-efficacy (Bernier et al., 2010). In an autonomy supportive context, children are more likely to take initiative, engage in problem solving, and persist on tasks, helping build their working memory and inhibitory control skills.

In comparison, supportive presence was only significantly related to improvements in child working memory. Prior studies with younger samples have found that supportive parenting in infancy is linked to stronger working memory skills at 3 years (Rhoades et al., 2011; Towe-Goodman et al., 2014). In a supportive parenting context, children will, on average, find it easier to maintain attentional focus and persist on tasks that may be stressful (Vandenbroucke et al., 2017). We extended prior findings by documenting that the positive effects of supportive parenting on child working memory can persist through early childhood years, until school entry.

Consistent with the early childhood coercion model (Scaramella & Leve, 2004), a transactional relationship was found between maternal hostility and child inhibitory control, with greater maternal hostility (in early childhood) predicting more inhibitory control difficulties in middle childhood, and poor inhibitory control (in early childhood) predicting more maternal hostility in middle childhood. Hostile forms of parenting model dysregulated behavior for the child, and elicit strong negative affect making it harder for children to engage in inhibitory control (Hughes & Ensor, 2006). Children who have difficulties with inhibitory control also elicit more hostile forms of parenting, thus creating a mutually reinforcing coercive cycle which hinders the growth of inhibitory control (Moilanen et al., 2010).

In terms of timing, as predicted, the effects of parenting behaviors on child EFs waned over time, such that parenting effects were significant only when examining change in EFs from early to middle childhood, but not from middle childhood to adolescence. This finding is in line with prior research that has found that parenting effects on child EFs are strongest in younger years (Belsky et al., 2007; KoşkuluSancar et al., 2023; Valcan et al., 2018). As such, parenting interventions targeting child EFs should focus on younger years, when the impact of specific parenting behaviors (e.g., autonomy support) is the strongest.

Contrary to our hypothesis, the lagged effects of parenting behaviors on child outcomes were only significant for child EFs. There was no lagged effect of parenting on child SR across the three time periods. This may be related to the self-reported nature of the child SR measure, which may not be as sensitive as the performance-based measures used to assess child EFs. Prior research has reported a lack of commonality between performance-based and self-report measures of EFs and SR (Cyders & Coskunpinar, 2011; Friedman & Banich, 2019), with performance-based measures being more sensitive to environmental inputs, including intervention effects, and self-report measures being more strongly related to real-world behavior (Demidenko et al., 2019). It is also possible that our measure of child SR was not as reliable as mothers may not be accurate reporters of their child’s SR abilities, especially in younger years. As children get older, mothers are better able to assess child’s SR across contexts, while lab-based tasks are limited in their ecological validity as compared to self-report measures (Ten Eycke & Dewey, 2016; Wallisch et al., 2018). We found this evidence in our transactional models, where the effects of child SR became more prominent in later years. It is also possible that shared method variance accounted for the stronger association observed between parenting and child EF since both observed parenting and lab

EF tasks were performance-based assessments. Nevertheless, future research should evaluate the developmental associations between specific parenting behaviors and child SR using more objective, performance-based measures of SR, as well as more comprehensive measures of SR unlike the current measure that focused on self-control dimension of SR.

### Child Effects on Parenting

We had predicted that child effects, especially those related to child SR on parenting would become more prominent in later years (middle childhood and beyond) as has been documented previously (Wang et al., 2013). In line with our hypothesis, we found child SR in middle childhood to be a significant predictor of parenting (specifically, supportive presence) in adolescence, controlling for stability pathways. Children whose mothers reported greater child SR in middle childhood showed an increase in supportive presence form of parenting from middle childhood to adolescence. Meta-analytic evidence corroborates child SR abilities predicting subsequent parenting behaviors across adolescence (Li et al., 2019). In our models, we found child SR only predicted supportive presence, and not autonomy support or hostility. One reason for this could be that it is easier for a parent to provide positive support when a child is well regulated. Other parenting behaviors (e.g., autonomy support) may be more strongly related to child EFs than child SR.

Unlike child SR effects which manifested in later years, the reciprocal effects of child EFs on parenting were observed in younger years, with inhibitory control difficulties in early childhood predicting more hostile parenting in middle childhood, controlling for stability pathways. On average, children who have difficulties with impulse control tend to make greater demands on parents’ time and skills and challenge their patience, and thus are more likely to elicit hostile parenting (Xing et al., 2021), with less warmth and greater intrusiveness (Merz et al., 2017). No other child effects were observed. Child EFs did not reciprocally predict supportive presence or autonomy support. We also did not observe any late-emerging effects of early parenting on child outcomes.

### Indirect Effects of Parenting on Child SR

Contrary to our hypothesis, there was no indirect effect of parenting on child SR, as mediated by child EFs. EFs underlie self-regulated behaviors and are expected to predict child SR differences (e.g., Khurana et al., 2018). The null effect is possibly related to measurement differences and lack of sensitivity in our self-reported measure of child SR. Prior studies have similarly reported low correlations between performance-based (EF) tasks and self-reported measures of SR (Cyders & Coskunpinar, 2011).

### Adolescence-Specific Associations Between Parenting and Child Outcomes

After accounting for the bidirectional linkages between parenting and child outcomes across time, we found significant residual covariances between autonomy support and child SR, and between supportive presence and child SR in adolescence, suggesting that parenting and child SR have unique associations in adolescence, not explained by early enduring, lagged, or late emerging effects. These effects were significant only in case of child SR, not child EFs. Reciprocal effects related to child EFs, when present, seemed to operate more strongly in younger years. In comparison, child SR effects appear to manifest in middle childhood-to- adolescent years, with stronger child SR in middle childhood being associated with increases in supportive presence from middle childhood to adolescence. Future research should examine the effects of specific parenting dimensions such as autonomy support and positive support on changes in self-regulation across adolescence. There may be other factors that explain the significant residual covariance. For instance, parents who are autonomy supportive and provide positive support may also engage in other parenting behaviors like rule-setting and monitoring, as well as model well-regulated behavior and structure the child’s environment (e.g., routines) all of which is linked to better SR in adolescence (Laible et al., 2015). Future work should examine the effects of other specific parenting behaviors on change in SR during adolescence.

There was no significant residual covariance between child SR and hostility in adolescence. This could be because as children grow older they become better regulated and thus elicit less hostile parenting. It could also be that there is less variance in hostility in the current sample, making it difficult to detect a significant residual covariance. Overall, while parenting has significant associations with child SR in adolescent years, the associations of parenting behaviors with child EFs are clearly limited to early-to-middle childhood years.

### Limitations

The following limitations of the study should be considered. First, we were not able to account for genetic effects, which may explain some or all of the associations observed between parenting behaviors and child outcomes. This limitation could be addressed in future work with other datasets that are able to utilize family designs, such as children of twins and sibling fixed-effect studies, to test impacts of parenting behaviors on child outcomes while accounting for genetic influences.

Second, we focused on maternal parenting behaviors only because there was less missingness across the waves in maternal parenting data. Fathers play an important and unique role in influencing child outcomes (Lucassen et al., 2015), and future research should examine these effects, including similarities and differences in maternal and paternal parenting behaviors and their impact on child outcomes.

Third, even though most of the measures used in the present study were objective and performance-based, for child SR we utilized maternal self-reports of child selfcontrol because this measure was assessed repeatedly across the waves, and thus conducive to RI-CLPM. The other variable option for child SR in the SECCYD data was delay of gratification, and this variable was not assessed across multiple time points. Further, although delay of gratification provides a “performance-based” assessment of a dimension of child SR, it is highly context-dependent and may not a robust predictor of long-term outcomes (Watts et al., 2018). Relatedly, we could not include cognitive flexibility – the third core component of EF – in our models because this EF dimension was not consistently assessed across all years in the SECCYD study.

Fourth, for our analyses, we had to collapse multiple assessments for some of the measures (e.g., parenting, child SR) across the same developmental period (e.g., 24-, 36-, and 54-month assessments were combined for “early childhood”). This may have reduced some of the variance in our measures; however, this step was necessary to model transactional linkages using RI-CPLMs which provide a much more stringent test of causal associations accurately accounting for between and within-person differences.

Fifth, our analysis approach may have generated results that differ from those reported in previous studies. Findings generated by between-person analytic approaches (e.g., correlational tests in a cross-sectional design) and potentially flawed approaches that conflate between- and within-unit variances (e.g., CLPM) may not be consistent with findings generated by analyses that disentangle the different sources of variation (Hamaker et al., 2015; Lucas, 2023). Finally, the sample for the SECCYD study was not sociodemographically and socioeconomically diverse, making it difficult to examine differences across groups, and limiting the generalizability of findings.

While our study provides valuable insights into population-level transactional linkages between parenting behaviors and child EFs and SR across developmental periods, it is essential to exercise caution when interpreting these results at the individual-level. The population-level associations we reported may not translate to correlations at the level of individual persons. Several factors may contribute to this distinction, including the ecological fallacy, Simpson’s paradox, and non-ergodicity (Lundh, 2023).

### Conclusion

This study highlights the important but nuanced relationships between parenting behaviors and child cognitive and self-regulatory outcomes. As predicted, parenting behaviors have cascading effects on child outcomes throughout development: parents who cultivate children’s autonomy and maintain a supportive presence early on in their lives raise children with stronger executive functioning in middle childhood. Autonomy support in particular may be important to target in parenting interventions and prevention efforts given the breadth of its effects on multiple components of child EF. However, children also affect their parents: early difficulties with inhibitory control may evoke hostile parenting in middle childhood, indicating a need for targeted interventions for parents of children who demonstrate limitations in this area. Such programs could provide parents with skills-based training to develop and maintain patience, warmth, and sensitivity when faced with strains on their time and attention due to children who behave impulsively. Because late-emerging and adolescent-specific effects of parenting behaviors on child EFs were limited in our study, our findings point to the importance of early intervention to promote positive and effective parenting. Future research should investigate how specific dimensions of parenting are related to self-regulatory outcomes in adolescent years. Identifying mechanisms of effects of parenting on child outcomes, as well as policies and practices that promote positive and supportive parenting should also be prioritized in future research.

## Data Availability

Data and materials from the NICHD SECCYD are available online: icpsr.umich.edu/web/ICPSR/series/233.

## Action editor

Lars-Gunnar Lundh served as action editor for this article.

## Open access

This article is distributed under the terms of the Creative Commons Attribution 4.0 International License (http://creativecommons.org/licenses/by/4.0/), which permits unrestricted use, distribution, and reproduction in any medium, provided you give appropriate credit to the original author(s) and the source, provide a link to the Creative Commons license, and indicate if changes were made.
